# Use of silkworms for identification of drug candidates having appropriate pharmacokinetics from plant sources

**DOI:** 10.1186/1471-2210-10-7

**Published:** 2010-06-11

**Authors:** Yukihiro Asami, Ryo Horie, Hiroshi Hamamoto, Kazuhisa Sekimizu

**Affiliations:** 1 Department of Microbiology, Faculty of Pharmaceutical Sciences, Graduate of the University of Tokyo. 7-3-1, Hongo, Bunkyo-ku, Tokyo, Japan

## Abstract

**Background:**

We use silkworms to evaluate therapeutic effects of drug candidates. Our previous reports have revealed that there are common mechanisms of pharmacokinetics of chemicals in silkworms and mammals. In this report, we attempt to establish a method by using silkworms to identify chemicals from plant extracts which are absorbed from intestine and also stably exist in body fluids.

**Results:**

Three compounds were detected in the silkworm hemolymph by HPLC analysis after midgut injection of acetone extracts of seihi, an herbal medicine obtained from orange peel. Analyses with MS and NMR revealed that the compounds were nobiletin, heptamethoxyflavone, and tangeretin. These compounds are reported to be stable in mammalian blood. The half-life of each of these compounds in the silkworm hemolymph was 18, 26 and 34 h, respectively.

**Conclusions:**

These findings suggest that silkworms can be used as a model animal to easily identify compounds with appropriate pharmacokinetic behavior.

## Background

Appropriate behavior in human bodies from the view of pharmacokinetics is an essential feature for drug candidates, since it is needed for therapeutic effects of the compounds [1]. In fact, most drug candidates screened by *in vitro *systems have problems in pharmacokinetics, and therefore, they do not show therapeutic effects in disease models with animals [2]. Mammals like mice and rats are chosen as model animals. A problem is that it is not easy to examine pharmacokinetics of a large number of drug candidates at early stages of drug development, since a huge amount of financial cost are needed to take care of mammals in laboratory facilities. It is also pointed out that sacrificing a large number of mammals causes ethical problems from a view of animal protection. The latter point is coming to be serious so that it is going to be a major factor to slow down the speed of drug development in industrialized countries. To solve these problems, use of invertebrates, which can be used with a large numbers with low costs, is desired [3].

We are currently proposing usefulness of silkworms to evaluate therapeutic effects of drug candidates. The size of silkworms is large enough to handle so that one can easily inject fixed volume of sample solution with syringes into hemolymph, a blood of silkworms. Two distinguishable protocols for injection of sample solution are available for silkworms; *i.h*. (intra hemolymph) and *i.m*. (intra midgut) injections. The former corresponds to injection into vein (*i.v.*), the latter to oral administration (*p.o*.) in humans. Pharmacologic experiments with isolated organs like midgut and fat body, which correspond to intestine and liver of mammals, are possible with silkworms. Those techniques are not easily applicable for fruit fly and nematoda, which are recently used as model animals for diseases, due to their small bodies. We previously reported establishment of infection models of silkworm with pathogenic bacteria and true fungi [4]. We demonstrated that the values of ED_50_, amounts of antibiotics needed for therapeutic effects to 50% of animals, and LD_50_, amounts of chemicals needed to kill 50% of animals, by the fixed amount of body weight of animals are similar between silkworms and mammals [5]. We also showed that silkworms have metabolic pathways with cytochrome P450s and conjugation enzymes [6]. It is also shown that the results of whether antibiotics can be absorbed from intestine or not are similar in silkworms and mammals [7]. These results suggest that there are common mechanisms of pharmacokinetics of chemicals between silkworms and mammals.

In this report, we describe our attempts to establish a method by using silkworms to identify chemicals from plant extracts which are absorbed from intestine and also stably exist in body fluids. We injected samples into midgut of silkworms, which corresponds to intestine in mammals, and examined substances that were stably exist in hemolymph. We identified three substances and determined their structures. We further confirmed that those substances stably exist in hemolymph of silkworm. To our knowledge, this is the first report proposing a method to identify chemicals possessing appropriate futures in pharmacokinetics.

## Results

### Detection of the compound which are absorbed from intestine and also stably exist in body fluids

**Figure 1:**
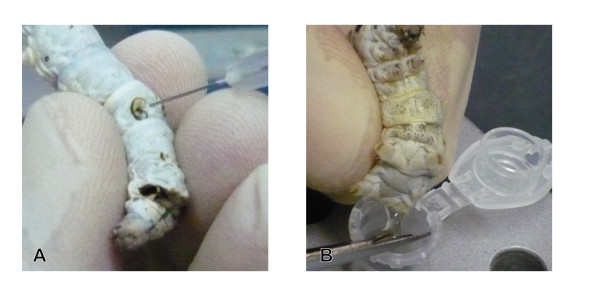
**Methods for injecting and harvesting hemolymph**. A: Injection of samples into silkworm midgut; B: Harvesting hemolymph from legs of silkworm.

**Figure 2:**
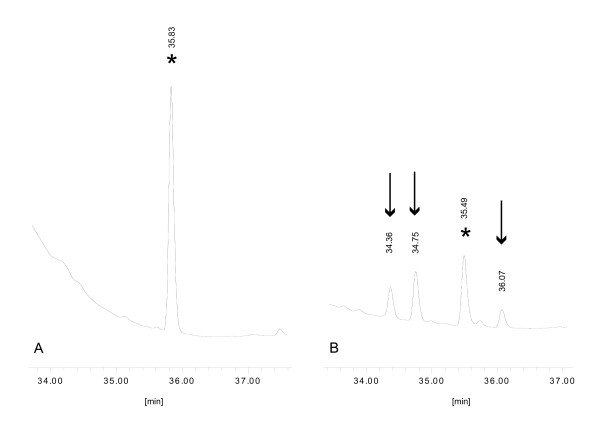
**Detection method of compounds in silkworm hemolymph by HPLC**. A) Magnified chromatogram of hemolymph of silkworm injected with saline. B) Magnified chromatogram of hemolymph of silkworm injected with an acetone extract of seihi. Asterisks indicate peaks from silkworm hemolymph. Arrows indicate peaks of specifically identified substances.

Firstly, we performed that acetone extract (500 μl) of seihi, an herbal medicine of orange peel from *Citrus reticulata Blanco*, was injected into intestine of silkworm (Figure 1A). After 6 hrs incubation, hemolymph was harvested from legs of larvae (Figure 1B). An equal volume of acetone was added, mixed vigorously, centrifuged, and the supernatant was analyzed by HPLC. By comparison of HPLC chromatogram obtained by hemolymph of silkworm injected with the acetone extract of seihi and that with saline (Figure 2A), 3 peaks with 34.36 min, 34.75 min, and 36.07 min were identified as a material from seihi (Figure 2B). Whole profiles of chromatography for each sample are shown in additional file 1A and 1B. Those 3 compounds were found as peaks of HPLC chromatogram of acetone extract of seihi at 34-39 min (additional file 2A). They are also observed by HPLC chromatogram of hexane extract of seihi (additional file 2B). Therefore, those chemicals were originally present in the seihi extract, and stably exist in silkworm hemolymph after absorbed from midgut.

### Purification of the three compounds

We tried to purify the above three compounds in a large scale for structural determination. Seihi (100 g) was extracted with 50% acetone (2 L), and acetone was removed by evaporation. The fraction (1 L) was extracted with an equal volume of *n*-hexane, and further purified by preparative HPLC. The yields of the fractions of compound A, B, and C at this stage were 11.6 mg, 21.1 mg, and 23.5 mg, respectively. Each sample was further purified by preparative thin layer chromatography. The yields of the final fractions of compound A, B, and C were 8.5 mg, 16.3 mg, and 19.1 mg, respectively (additional file 3). Each fraction showed a single peak on HPLC (additional file 4).

### Determination of structure of the three compounds

**Figure 3:**
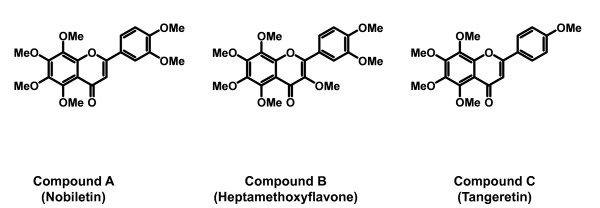
**Chemical structures**. Chemical structures of Compound A (Nobiletin), Compound B (Heptamethoxyflavone), and Compound C (Tangeretin).

We next try to determine structures of compound A, B, and C. Mass-spectrometry analysis revealed molecular masses of compound A, B, and C were 402, 432, and 372, respectively. The results of ^1^H-NMR spectrum measurement revealed the presence of hydrogen, characteristic methoxy, and aromatic ring protons. Based on comparison with previously published data for ingredients of seihi and compound A, B, and C measured both FAB-MS and ^1^H-NMR spectrum data [8,9] [Compound A (Nobiletin): FAB-MS m/z 403 (M+H)^+^; ^1^H NMR (500 MHz, CDCl_3_), δ_H _7.58 (dd, *J *= 2.0, 8.6 Hz, 1H), 7.42 (d, *J *= 2.0 Hz, 1H), 7.00 (d, *J *= 8.6 Hz, 1H), 6.62 (s, 1H), 4.11 (s, 3H), 4.03 (s, 3H), 3.98 (s, 3H), 3.97 (s, 3H), 3.96 (s, 6H). Compound B (Heptamethoxyflavone): FAB-MS m/z 433 (M+H)^+^; ^1^H NMR (500 MHz, CDCl_3_), δ_H _7.85 (dd, *J *= 2.0, 8.7 Hz, 1H), 7.81 (d, *J *= 2.0, 8.7 Hz, 1H), 7.02 (d, *J *= 8.7 Hz, 1H), 4.10 (s, 3H), 4.01 (s, 3H), 3.98 (s, 6H), 3.95 (s, 3H), 3.89 (s, 3H). Compound C (Tangeretin): FAB-MS m/z 373 (M+H)^+^; ^1^H NMR (500 MHz, CDCl_3_), δ_H _7.88 (d, *J *= 8.6 Hz, 2H), 7.03 (d, *J *= 8.6 Hz, 2H), 6.61 (s, 1H), 4.11 (s, 3H), 4.03 (s, 3H), 3.95 (s, 6H), 3.89 (s, 3H).],we concluded that compound A, B, and C were Nobiletin (C_21_H_22_O_8_, MW = 402), Heptamethoxyflavone (C_22_H_24_O_9_, MW = 432), and Tangeretin (C_20_H_20_O_7_, MW = 372), respectively (Figure 3)

### Pharmacokinetics of the three compounds in silkworm hemolymph

**Figure 4:**
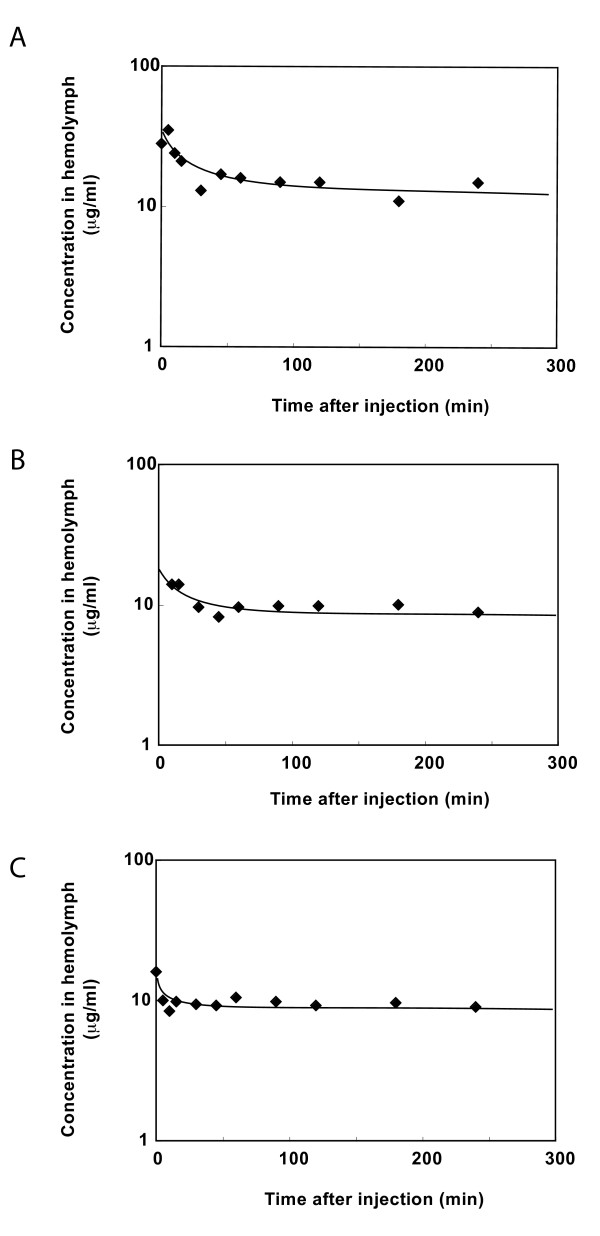
**Time course of decrease in concentrations of Nobiletin, Heptamethoxyflavone, and Tangeretin in silkworm hemolymph**. A: Nobiletin; B: Heptamethoxyflavone; C: Tangeretin

We assumed that their transport across midgut membrane might not be blocked by molecular mass barriers since the molecular masses of these compounds were smaller than 450[6]. We also assumed that compound A, B, and C were stable in hemolymph, since they were found in hemolymph of silkworm 6 hrs later after injection into midgut. To test the stability of the compounds in hemolymph, we injected purified compound A, B, and C into hemolymph of silkworm and examined their amounts in hemolymph after appropriate period of injection. The concentration of compounds A, B, and C in hemolymph was determined by HPLC. The results demonstrated that concentrations of compound A, B, and C in hemolymph decreased quickly soon after injection followed by slow decrease at the second stage. The half-lives of compound A, B, and C at the second stage were estimated to be 18, 26, and 34 hrs, respectively (Figure 4).

## Discussion

In this study, we demonstrated a method to identify chemical compounds that show appropriate pharmacokinetics as drug candidates by using silkworm, an invertebrate animal. We injected an acetone extract of seihi into midgut of silkworm, and identified three compounds that appear and stably exist in hemolymph. The half-lives of these compounds in hemolymph of silkworm were 18, 26, and 34 hrs, indicating their stability in hemolymph. Structural analysis demonstrated that they are polymethoxyflavones with the flavonoid skeletons. Those compounds were reported to have pharmacological actions of anti-inflammation, anti-oxidation, and anti-malignancy[9-12]. Since they show interesting physiological activities, researches examined pharmacokinetics of the compounds in mammals. For instance, good characters as drug candidates of pharmacokinetics of nobiletin and tangeretin in rats has been reported[9,13-15]. Half-life of nobiletin in mouse blood was suggested to be longer than 24 hrs[14]. This means that those three compounds identified as chemicals that show appropriate pharmacokinetics in silkworms are compounds whose pharmacokinetics is appropriate in mammals. In addition, we used this method to identify some compounds in soil bacteria culture supernatant that exhibit stable pharmacokinetics in silkworm hemolymph and are therefore appropriate for further screening (manuscript in preparation).

An evaluation method of pharmacokinetics for a large number of chemicals can be applied not only to plant extract, but also to other natural sources including microorganisms and animals. The method is also useful to identify such chemicals from chemical libraries containing a large number of synthetic organic compounds. The most characteristic advantage of the method by using silkworm is that one can inject test samples into both hemolymph, a blood of larvae, and midgut, that corresponds to intestine of mammals. The former corresponds to intra vein injection, and the latter corresponds to oral administration in humans. We previously reported that there are common features in barriers by molecular mass of midgut of silkworm and intestine of mammals[6]. Taken together with our present findings, a method by using silkworms seems to be useful to screen drug candidates that are effective by oral administration.

## Conclusions

Appropriate features in pharmacokinetics are essential for medicine to show therapeutic effects in disease models with animals. Importance of availability by oral administration should be emphasized since it decreases the burden of human patients. A problem is that the most of compounds that adsorbed from intestine are unstable in blood, so that unable to show therapeutic effects. Therefore, to get drug candidates that show appropriate pharmacokinetics, a long period and a high cost should be consumed. It should be also pointed out that a large number of mammals have been sacrificed for evaluation of pharmacokinetics of drug candidate, and arguments of ethical issues from the view of animal protection have been arisen. Use of silkworm to evaluate pharmacokinetics of chemicals will be a clue to overcome those problems.

## Methods

### Animals

Fertilized eggs of silkworm (Hu•Yo × Tsukuba•Ne) were purchased from Ehime Sanshu (Ehime, Japan). Hatched larvae were reared at 27 °C with Silkmate 2S (Nihon Nosan Kogyo, Tokyo, Japan), an artificial diet for young silkworms to 4^th ^instar. Larvae of 5^th ^instar were fed with Chisan 2-rei (Katakura Kogyo, Tokyo Japan), an artificial diet for silkworms at the last stage. Humidity and light cycle were not controlled.

### Chromatography

HPLC analysis was performed by a column of PEGASIL ODS (4.5φ × 250 mm) with isocratic elution of 10% CH_3_CN for 15 min, followed by gradient elution of 10%-100% CH_3_CN for 40 min, and then 100% CH_3_CN 15 min at the flow rate of 1 ml/min. Preparative HPLC was carried out by a column of PEGASIL ODS (20φ × 250 mm) with isocratic elution of 40% CH_3_CN at the flow rate of 9 ml/min. Preparative thin layer chromatography was done with 25 HPTLC plates RP-18 F_254S _(Merck) with a developing solution of hexane and ethyl acetate (3:1), or with Silica gel 60F_254 _(Merck) with a developing solution of chloroform and methanol (2:1).

### Structural analysis

Structures of chemicals were analyzed by ^1^H-NMR or FAB-MS (low resolution fast atom bombardment mass spectrometry), and their spectrometric data were analyzed and compared with the results reported previously. ^1^H-NMR spectrometry was performed with a JEOL EC-500 spectrometry meter. Samples were dissolved in CDCl_3 _(Merck, NJ, USA). FAB-MS spectrometric data was obtained with a JEOL JMS-700 mass spectrometer (JEOL). Glycerol (Sigma) was used as a matrix.

### Determination of chemicals in hemolymph of silkworms

A sample solution (50 μl) was injected into hemolymph of 5^th ^instar larvae (1.8-2.0 g) with a syringe. Hemolymph was harvested from legs and mixed with an equal volume of acetone, followed by centrifugation at 15, 000 rpm for 5 min at 4 °C. Supernatant was dried up, dissolved into 50% methanol to prepare for an HPLC sample. Standard curves of each compound were drawn by measurement of peak area on HPLC charts, and the amounts of each compound in the sample were determined.

### Injection of samples into silkworms

Samples were injected as previously described (4). Briefly, for intra-midgut injection, samples were injected toward the center of the body cavity from the 5^th ^somite of the abdomen of a living silkworm using a 27-gauge needle. For intra-hemolymph injection, a 27-gauge needle was inserted parallel to the outer skin at the 5^th ^somite of the abdomen. The accuracy of these injection techniques was confirmed using trypan blue, which did not leak out from the midgut (additional file 5) [6]. Less than 1% of the injection sample was detected in the hemolymph immediately after the injection of compound C into the midgut (additional file 6).

## Authors' contributions

YA performed the purification and structural analysis. RH analyzed the pharmacokinetics. HH participated in designing the study. KS conceived the study and coordinated the writing of the manuscript. All authors read and approved the final manuscript.

## Supplementary Material

Additional file 1 **Profiles of hemolymph of silkworm with HPLC analysis**. A) Chromatogram of hemolymph of silkworm injected with saline into midgut. B) Chromatogram of hemolymph of silkworm injected with an acetone extract of seihi into midgut. Analysis condition of HPLC: PEGASIL ODS (4.5φ × 250 mm) with isocratic elution of 10% CH_3_CN for 15 min, followed by gradient elution of 10%-100% CH_3_CN for 40 min, and then 100% CH_3_CN 15 min at the flow rate of 1 ml/min. Click here for file

Additional file 2 **Profiles of seihi extracts with HPLC analysis**. A) Chromatogram of an acetone extract of seihi. B) Chromatogram of a hexane extract from the acetone extract of seihi. Analysis condition of HPLC: PEGASIL ODS (4.5φ × 250 mm) with isocratic elution of 10% CH_3_CN for 15 min, followed by gradient elution of 10%-100% CH_3_CN for 40 min, and then 100% CH_3_CN 15 min at the flow rate of 1 ml/min. Click here for file

Additional file 3 **Isolation scheme of compounds A, B, and C**. Click here for file

Additional file 4 **Chromatogram of compounds A, B, and C**. HPLC analysis condition of compounds of A and B: PEGASIL ODS (4.5φ × 250 mm, 40% CH_3_CN isocratic, flow rate 1 ml/min). HPLC analysis condition of compound C: PEGASIL ODS (4.5φ × 250 mm, 45% CH_3_CN isocratic, flow rate 1 ml/min) Click here for file

Additional file 5 **Evaluation of injection techniques using trypan blue**. A) Intra-hemolymph injection of trypan blue solution. Blue color of the dye in the hemolymph is observed through the skin. B) Intra-midgut injection of trypan blue solution. The blue color of the dye cannot be seen, because the trypan blue did not leak out from the midgut [6]. Click here for file

Additional file 6 **Intra-midgut and intra-hemolymph injection of compound C**. A) HPLC analysis of hemolymph just after midgut injection of compound C B) HPLC analysis of hemolymph after intra-hemolymph injection of compound C. Click here for file
