# Supplementary figures and images for: Use of silkworms for identification of drug candidates having appropriate pharmacokinetics from plant sources
Source: BMC Pharmacol. 2010 Jun 11;10:7. doi: 10.1186/1471-2210-10-7 (PMC2898707; doi:10.1186/1471-2210-10-7)

**A**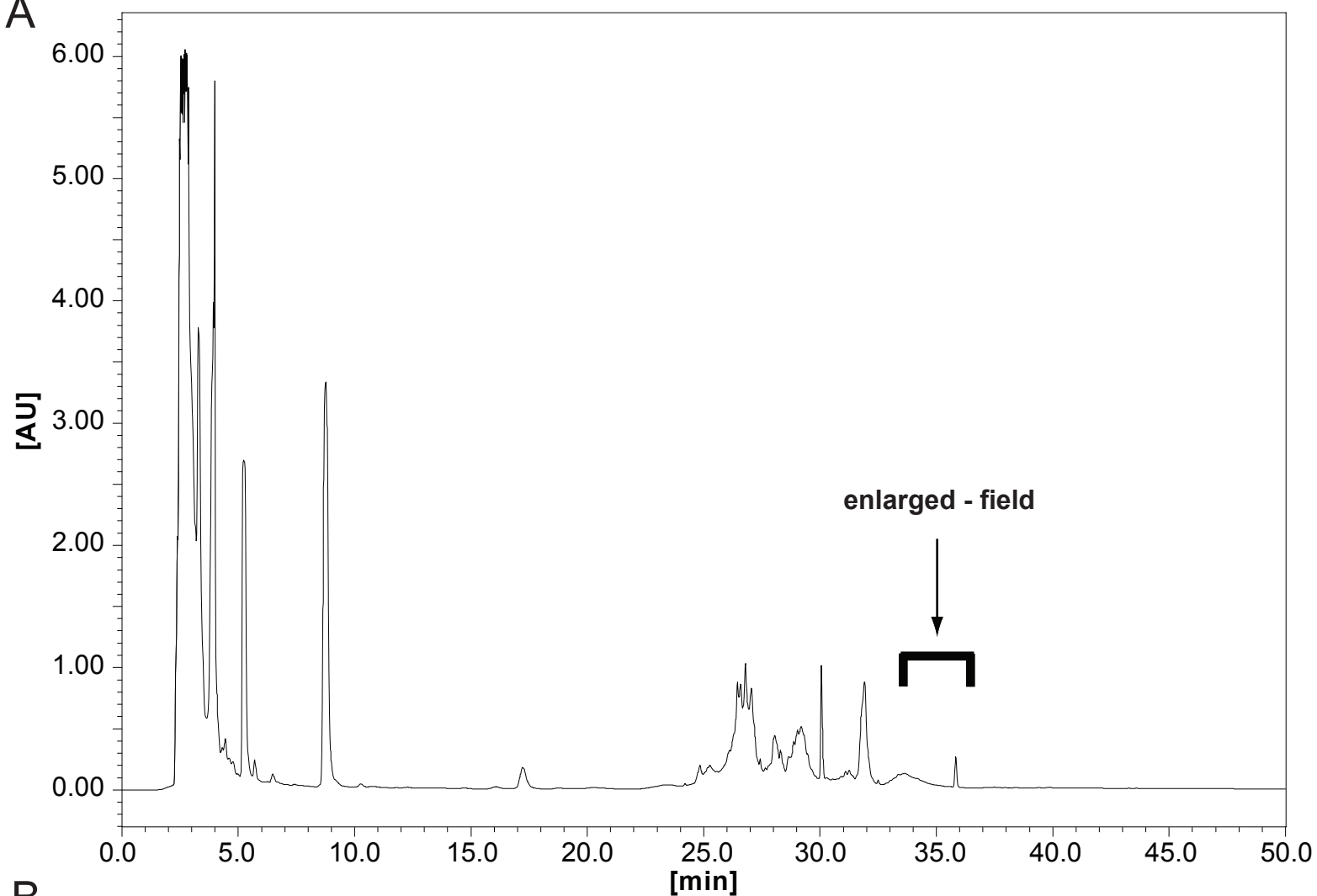**B**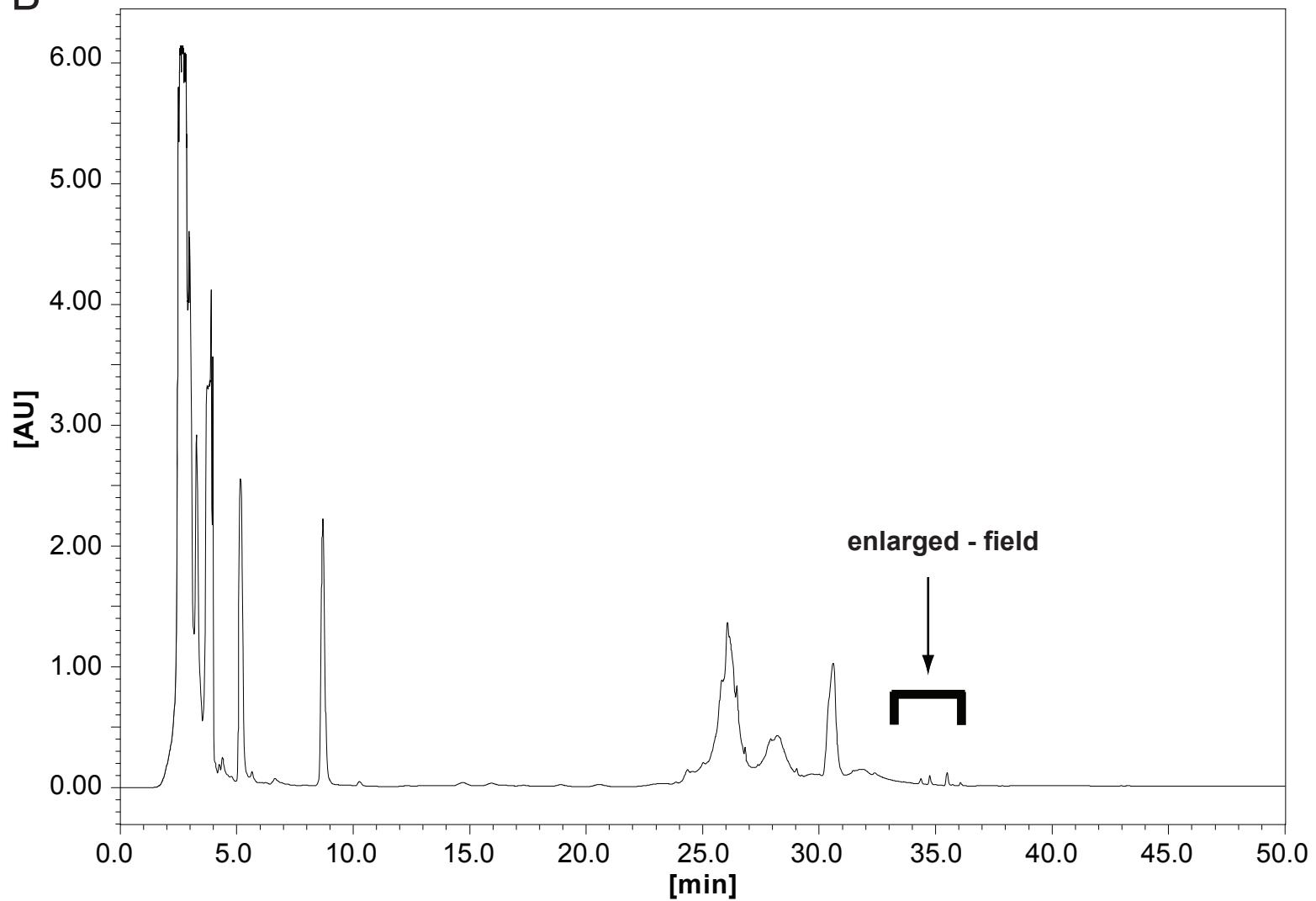

Supplement: Additional file 1 — Profiles of hemolymph of silkworm with HPLC analysis. A) Chromatogram of hemolymph of silkworm injected with saline into midgut. B) Chromatogram of hemolymph of silkworm injected with an acetone extract of seihi into midgut. Analysis condition of HPLC: PEGASIL ODS (4.5φ × 250 mm) with isocratic elution of 10% CH3CN for 15 min, followed by gradient elution of 10%-100% CH3CN for 40 min, and then 100% CH3CN 15 min at the flow rate of 1 ml/min. [file 1471-2210-10-7-S1.PDF]

**A**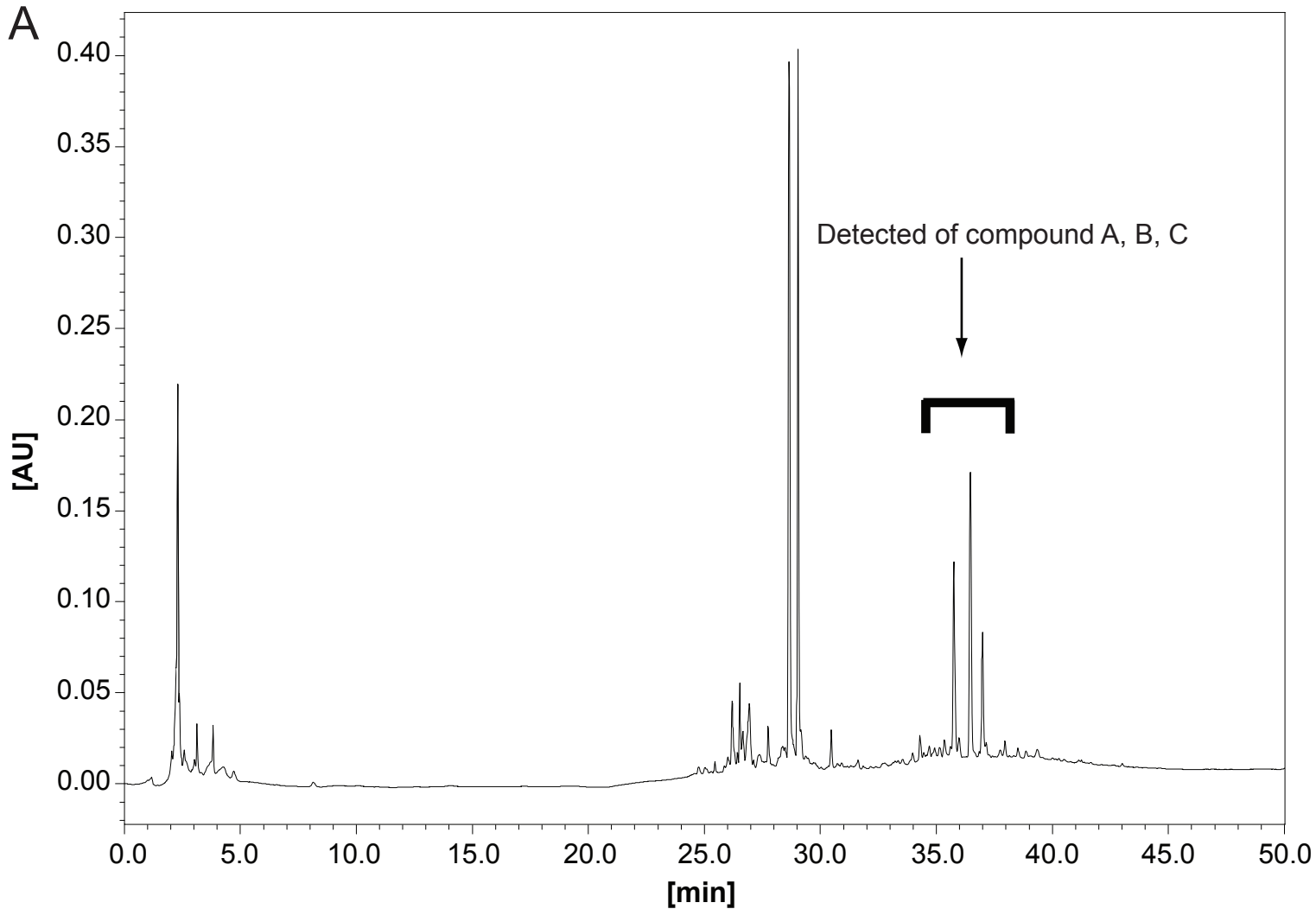**B**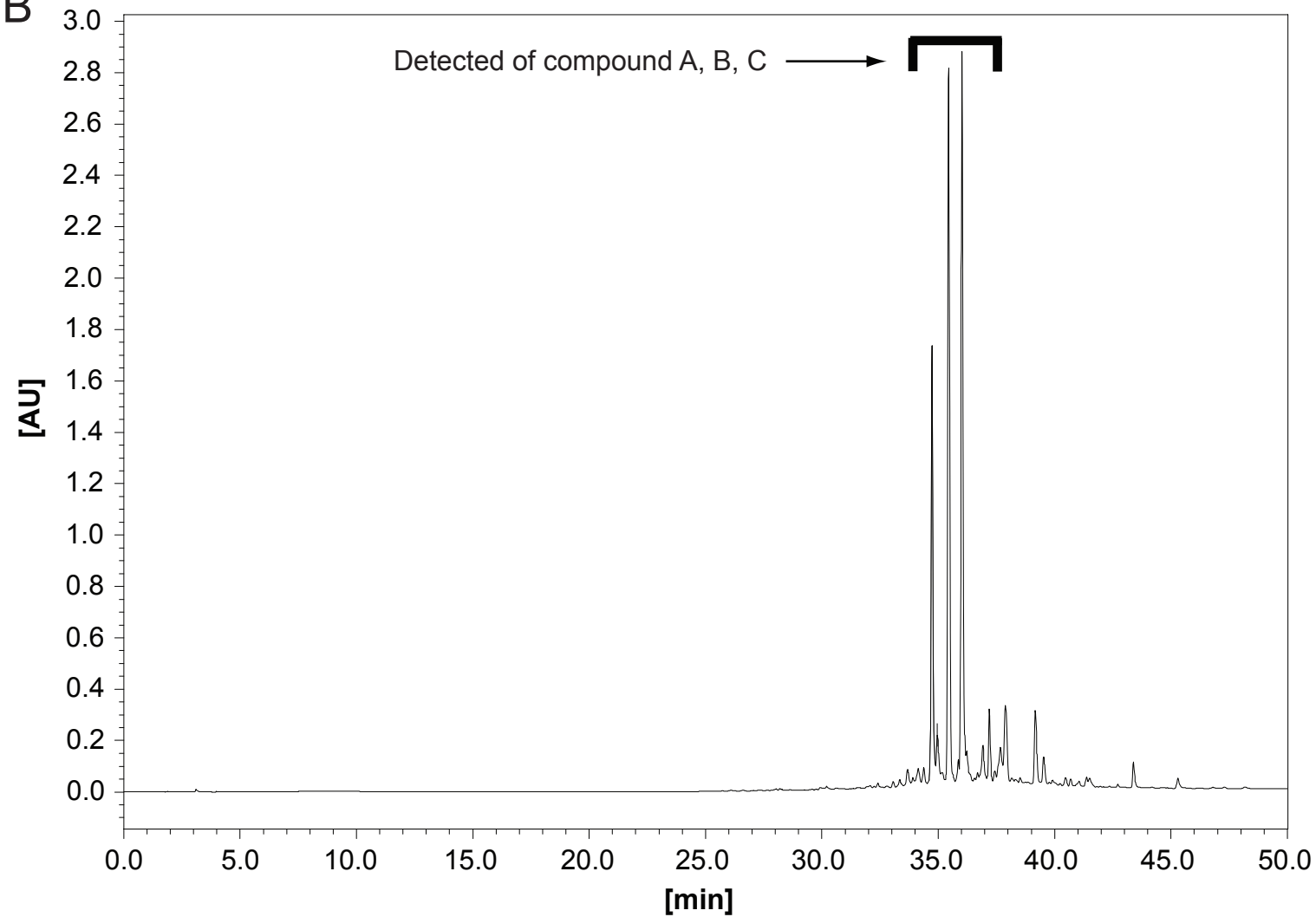

Supplement: Additional file 2 — Profiles of seihi extracts with HPLC analysis. A) Chromatogram of an acetone extract of seihi. B) Chromatogram of a hexane extract from the acetone extract of seihi. Analysis condition of HPLC: PEGASIL ODS (4.5φ × 250 mm) with isocratic elution of 10% CH3CN for 15 min, followed by gradient elution of 10%-100% CH3CN for 40 min, and then 100% CH3CN 15 min at the flow rate of 1 ml/min. [file 1471-2210-10-7-S2.PDF]

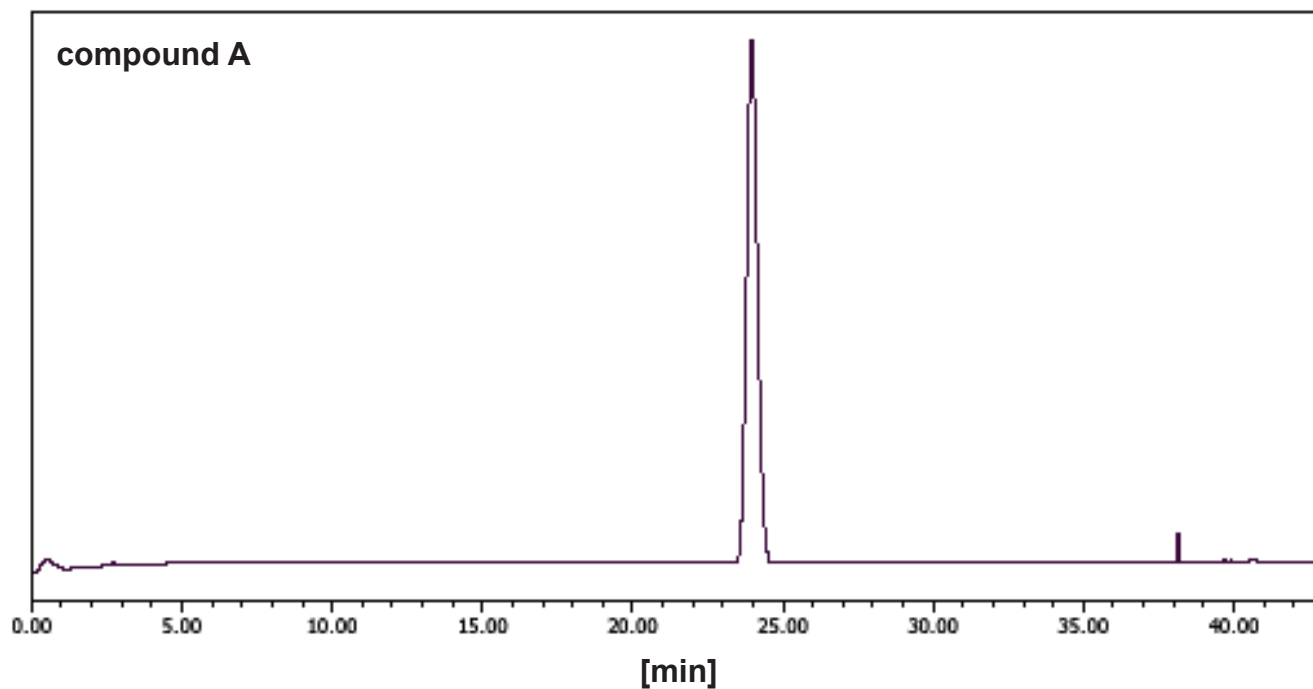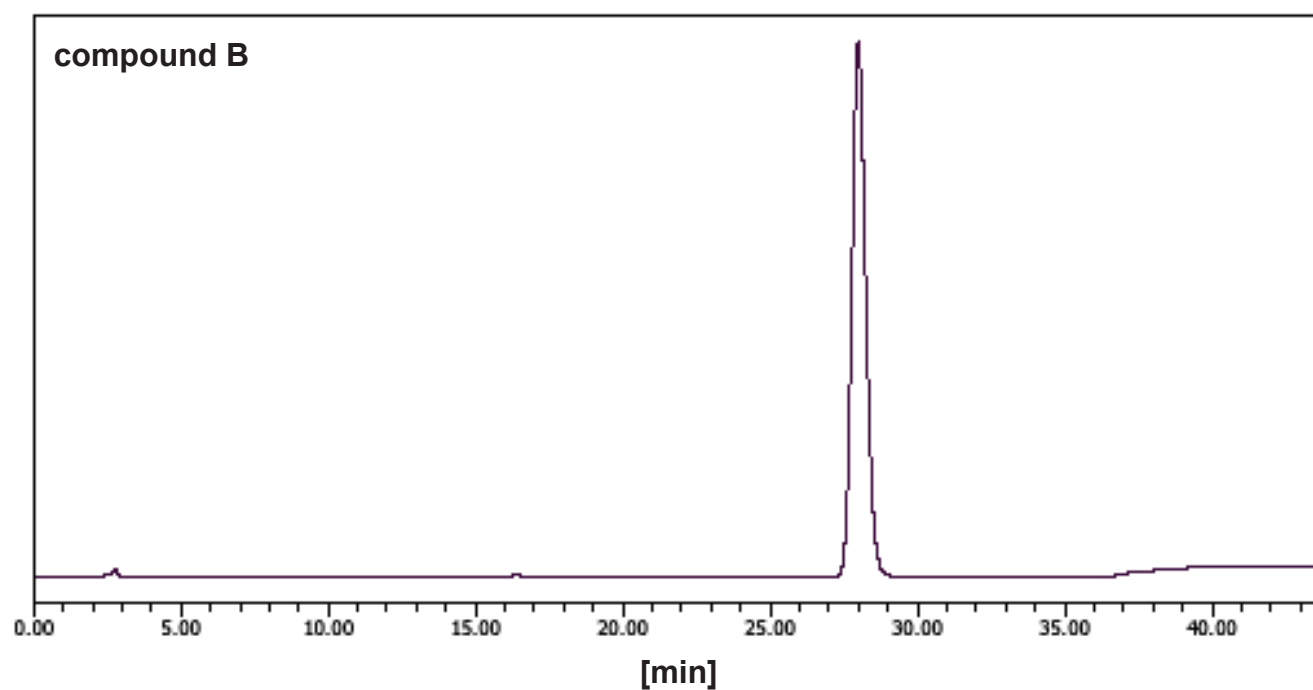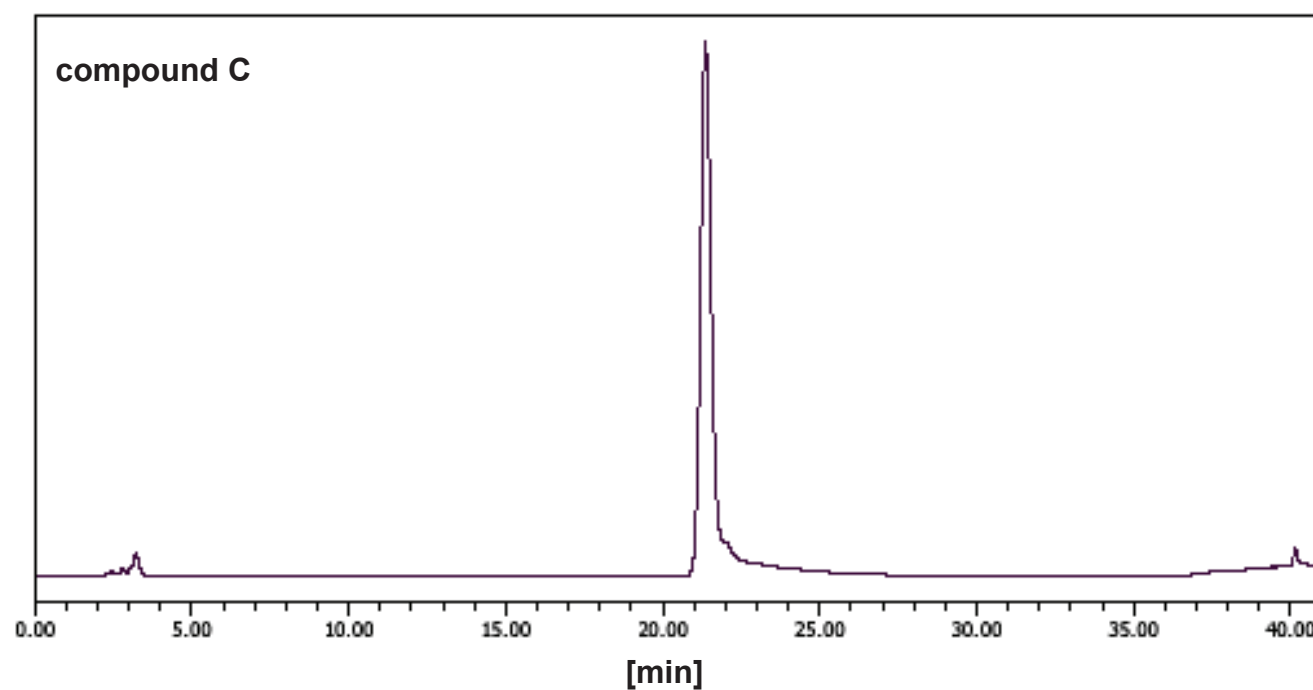

Supplement: Additional file 4 — Chromatogram of compounds A, B, and C. HPLC analysis condition of compounds of A and B: PEGASIL ODS (4.5φ × 250 mm, 40% CH3CN isocratic, flow rate 1 ml/min). HPLC analysis condition of compound C: PEGASIL ODS (4.5φ × 250 mm, 45% CH3CN isocratic, flow rate 1 ml/min) [file 1471-2210-10-7-S4.PDF]

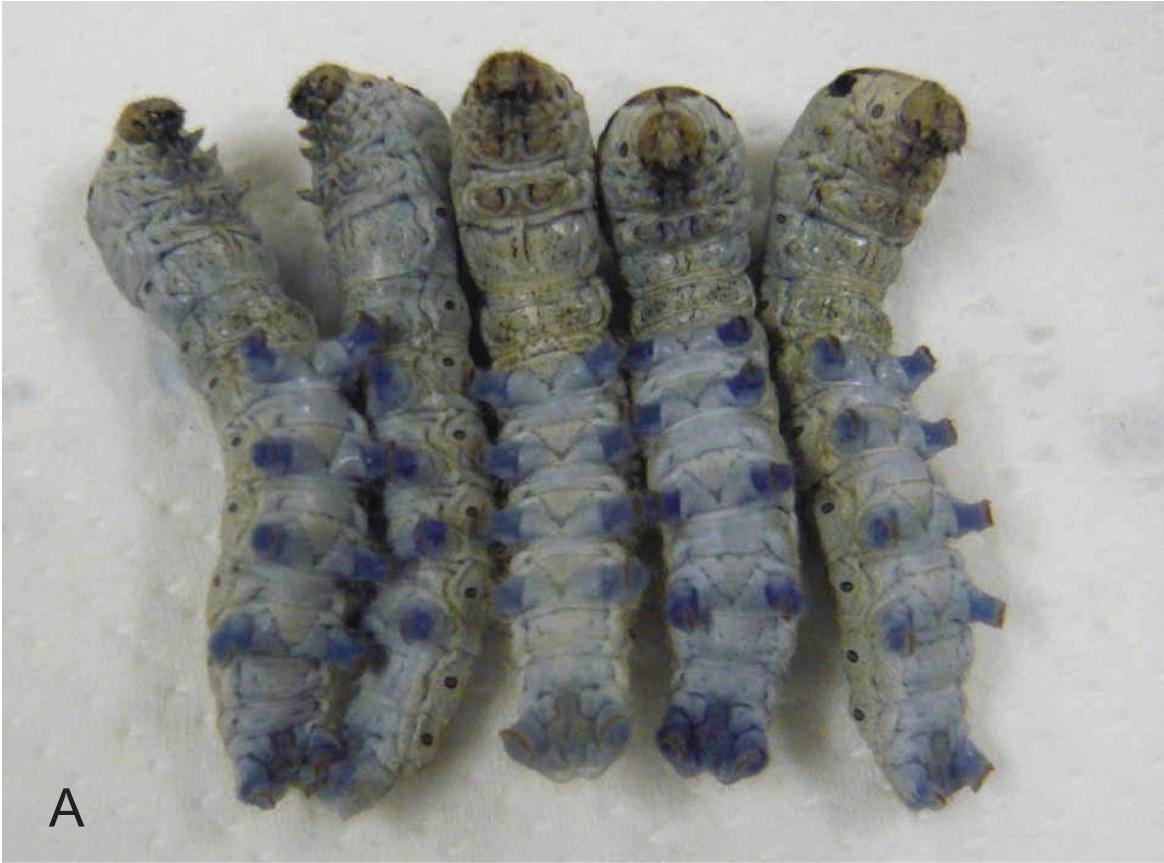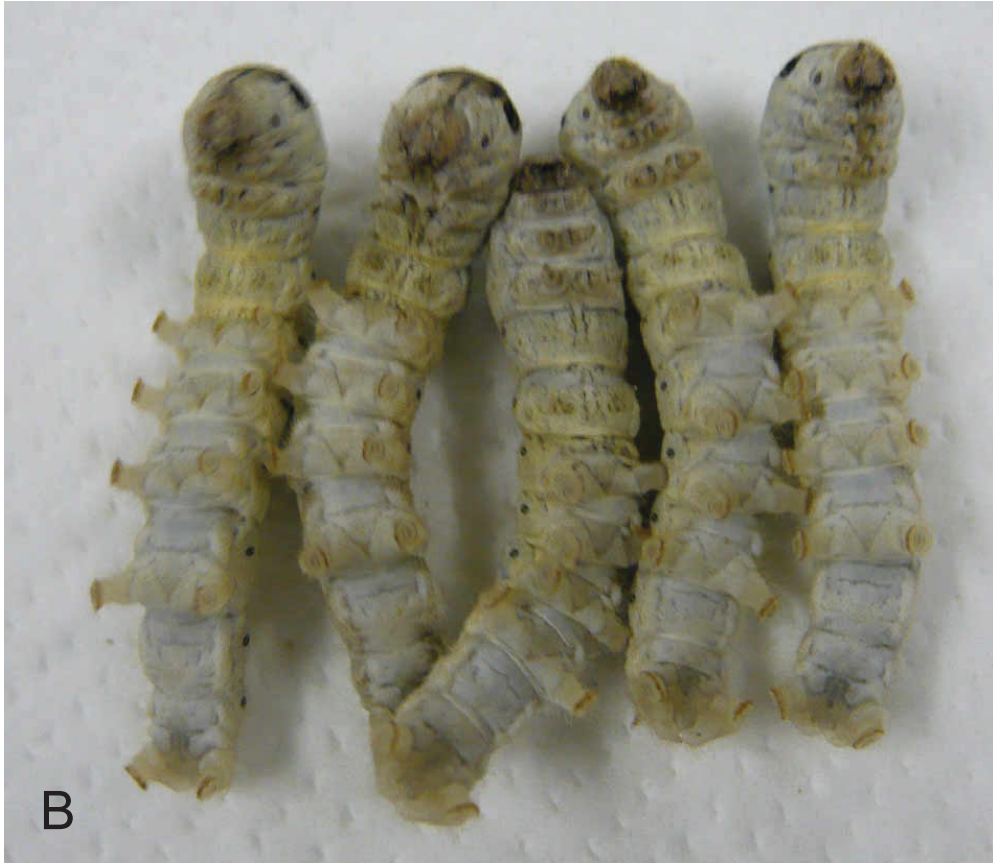

Supplement: Additional file 5 — Evaluation of injection techniques using trypan blue. A) Intra-hemolymph injection of trypan blue solution. Blue color of the dye in the hemolymph is observed through the skin. B) Intra-midgut injection of trypan blue solution. The blue color of the dye cannot be seen, because the trypan blue did not leak out from the midgut [6]. [file 1471-2210-10-7-S5.PDF]

**A**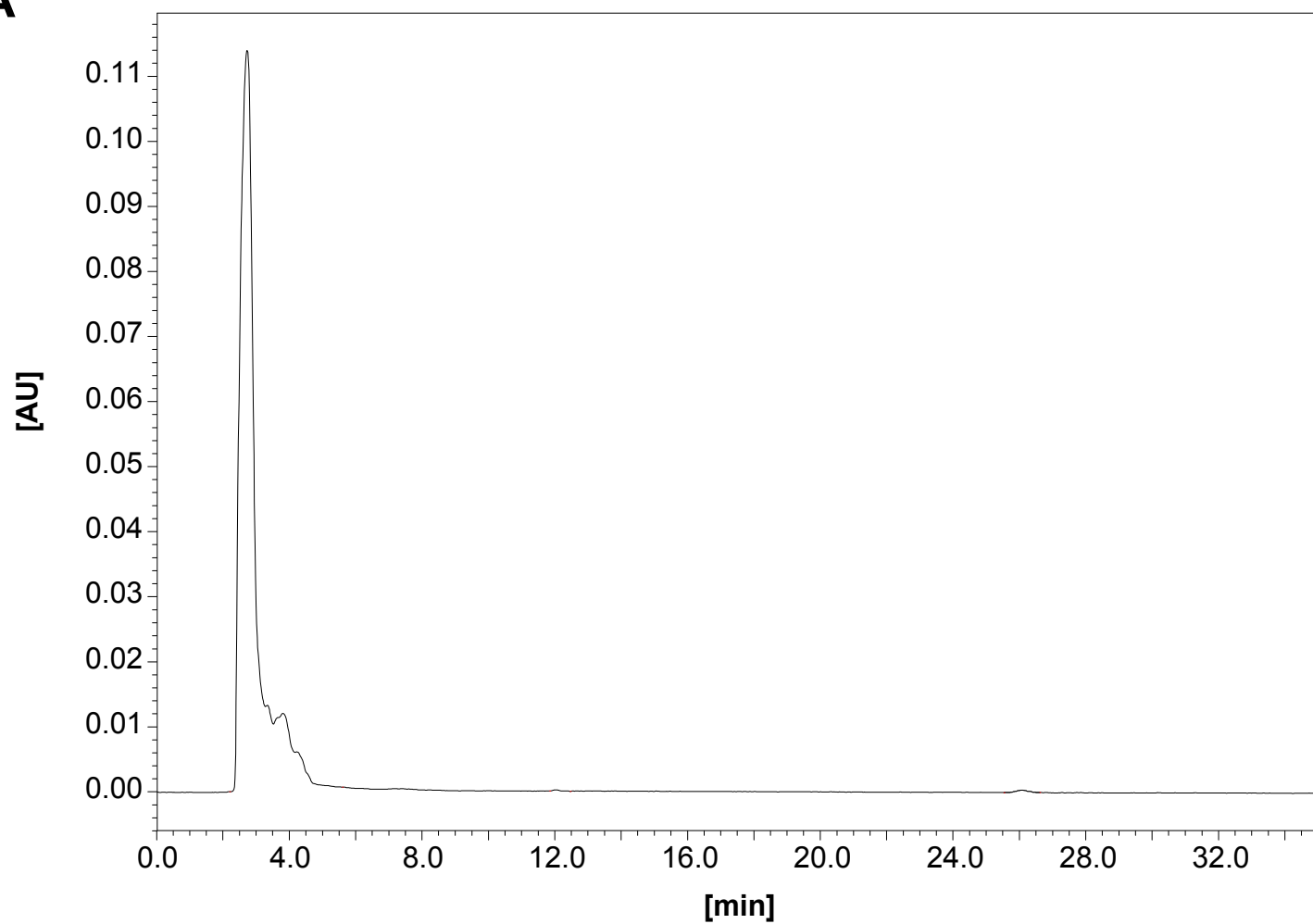**B**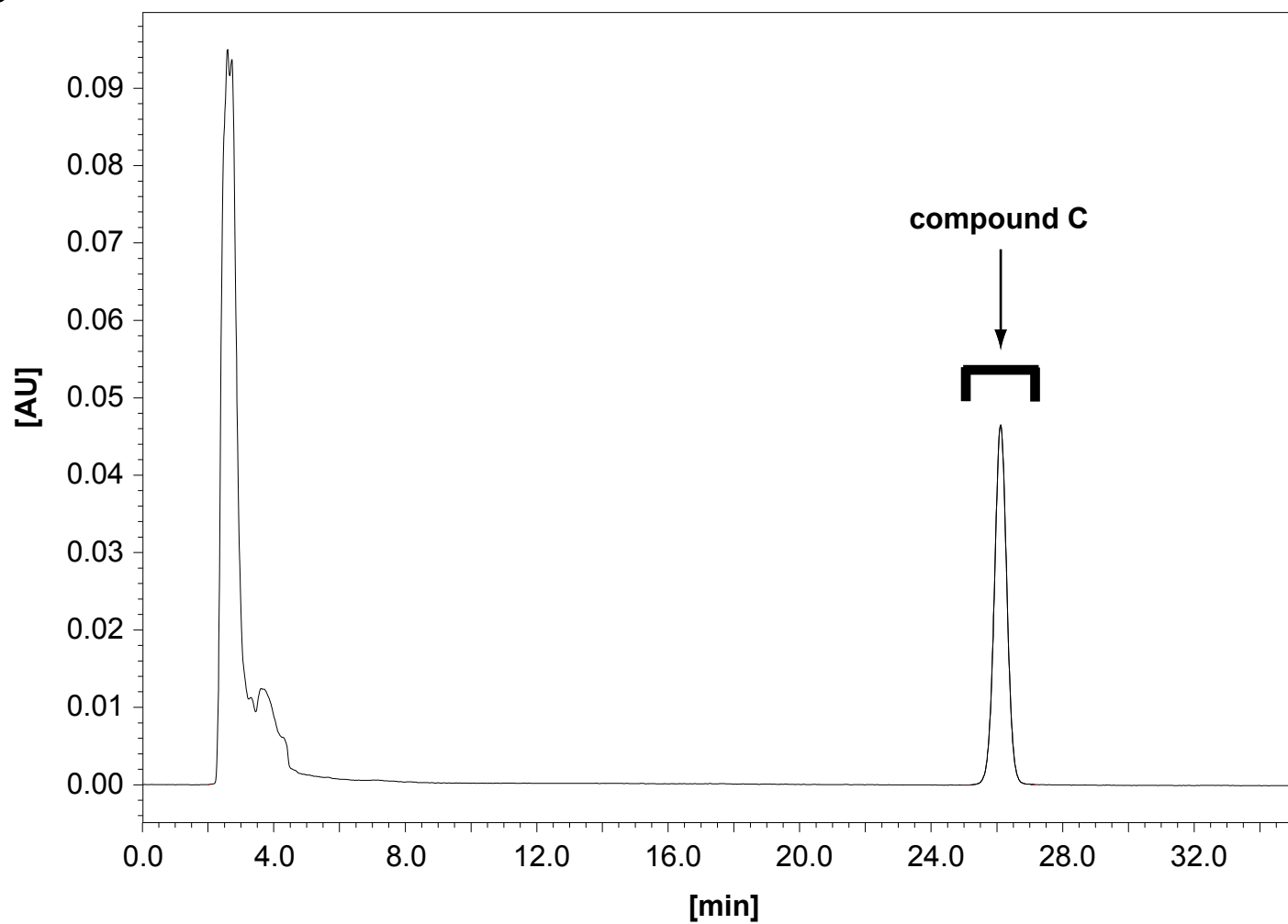

Supplement: Additional file 6 — Intra-midgut and intra-hemolymph injection of compound C. A) HPLC analysis of hemolymph just after midgut injection of compound C B) HPLC analysis of hemolymph after intra-hemolymph injection of compound C. [file 1471-2210-10-7-S6.PDF]
