# Supplementary material for: Use of silkworms for identification of drug candidates having appropriate pharmacokinetics from plant sources
Source: BMC Pharmacol. 2010 Jun 11;10:7. doi: 10.1186/1471-2210-10-7 (PMC2898707; doi:10.1186/1471-2210-10-7)
Supplement: Additional file 3 — Isolation scheme of compounds A, B, and C. [file 1471-2210-10-7-S3.PDF]

Citrus reticulata Blanco (100g)

extracted with 50% acetone aq. (2 liters)

filtrated with filter paper

concd. *In vacuo*

Water fraction (2 liters)

extracted with *n*-hexane (2 liters)

***n*-hexane fraction (2 liters)**

concd. *in vacuo*

Crude extracts (600 mg)

Prep. HPLC PEGASIL ODS (20 ϕ × 250 mm, 40% CH_3CN isocratic, 9 ml/min)

Crude fraction A (11.6 mg)

Prep. TLC (*n*-hexane / EtOAc =3 / 1)

HPTLC Patten RP-18

Fraction A (8.5 mg)

Prep. TLC (CH_3Cl / MeOH = 10 / 1)

Silica gel 60

Compound A (5.8 mg)

Crude fraction B (21.1 mg)

Prep. TLC (*n*-hexane / EtOAc =3 / 1)

HPTLC Patten RP-18

Fraction B (16.3 mg)

Prep. TLC (CH_3Cl / MeOH = 10 / 1)

Silica gel 60

Compound B (10.2 mg)

Crude fraction C (23.5 mg)

Prep. TLC (*n*-hexane / EtOAc =3 / 1)

HPTLC Patten RP-18

Fraction C (19.1 mg)

Prep. TLC (CH_3Cl / MeOH = 20 / 1)

Silica gel 60

Compound C (17.3 mg)
